# Beyond Mars and Venus: The role of gender essentialism in support for gender inequality and backlash

**DOI:** 10.1371/journal.pone.0200921

**Published:** 2018-07-24

**Authors:** Lea Skewes, Cordelia Fine, Nick Haslam

**Affiliations:** 1 School of Culture & Society, Interacting Minds Centre, Aarhus University, Aarhus, Denmark; 2 School of Historical & Philosophical Studies, The University of Melbourne, Victoria, Australia; 3 Melbourne School of Psychological Sciences, The University of Melbourne, Victoria, Australia; Institut Català de Paleoecologia Humana i Evolució Social (IPHES), SPAIN

## Abstract

It has been argued that gender essentialism impedes progress towards greater gender equality. Here we present a new gender essentialism scale (GES), and validate it in two large nationally representative samples from Denmark and Australia. In both samples the GES was highly reliable and predicted lack of support for sex-role egalitarianism and support for gender discrimination, as well as perceived fairness of gender-based treatment in the Australian sample, independently of two established predictors (i.e., social dominance orientation and conservative political orientation). In addition, gender essentialism assessed by the GES moderated some manifestations of the backlash effect: high essentialists were more likely to respond negatively towards a power-seeking female political candidate relative to a male candidate. Given the implications for possible workplace interventions, further work could usefully explore whether gender essentialism moderates other well-established forms of gender bias.

## Introduction

Expressions like “men are from Mars, women are from Venus” reflect essentialist thinking: the belief that all members of a category share fundamental or ‘essential’ qualities that make them what they are. Taking its cue from the philosophical claim that certain categories, dubbed ‘natural kinds’, possess such causal essences, a body of social psychological work has argued that laypeople often hold essentialist beliefs about social groups. Theorists and researchers [1–4] have proposed that essentialist thinking involves beliefs that a human group is natural, immutable, discrete, informative, historically and cross-culturally invariant, and grounded in deep-seated, biological, factors. Although some researchers have argued that psychological essentialism can sometimes implicate beliefs in social rather than biological determinism [5], the natural kind understanding of essentialism is dominant.

Gender is one of the first social categories we learn to apply [6]. It is strongly essentialized (e.g., [7]), and gender categories are often understood as biologically based ‘natural kinds’ [2, 8]. In line with this, contemporary arguments in favor of greater gender equality often draw on gender essentialist assumptions that women and men are distinctly, immutably, and naturally different, and thus have complementary skills to bring to the workplace. For example, differences between the sexes are sometimes described in categorical ways, and attributed in a deterministic fashion to fixed biological factors (see [9, 10]). Within the academic literature, social science research seeking to test the so-called ‘business case for gender diversity’, by testing for correlations between female leadership and firm profitability, is often implicitly gender essentialist, with the biological sex of a leader taken as a proxy for a fully enacted and distinctly feminine leadership style (see [11]).

However, contemporary science does not support an essentialist view of the sexes (e.g., [9, 12, 13, 14]). Moreover, theorists and researchers have proposed that essentialist thinking in general serves to justify existing social inequalities [4], rather than merely describing it neutrally. Contrary to the goals of gender equality advocates who put forward gender essentialist arguments, existing research provides reasons to anticipate that gender essentialist characterizations of the sexes will be associated with weaker endorsement of the desirability and feasibility of achieving greater gender equality in traditionally feminine and masculine roles. For example, Keller [15] found that greater endorsement of the deterministic role of biological factors in development was associated with higher levels of modern sexism, such as the belief that sexism is no longer pervasive and that progressive gender policies are unwelcome [16]. Similarly, exposure to biologically based theories of sex differences increases people’s acceptance of gender inequality [17]. Kray and colleagues recently found that the belief that gender roles are fixed (as opposed to changeable) is associated with greater gender system justification and preference for traditional roles, with biological attributions for gender differences and inequalities contributing to this association [18]. Gender essentialism (GE) has also been linked with enacted domestic arrangements and preferences supportive of traditional gender roles. Gaunt [19] found that fathers with more essentialist beliefs about parental roles were less involved with child care, independently of working hours and attitude toward the paternal role. More recently, Tinsley, Howell, and Amanatullah [20] found that greater endorsement of what they termed gender determinism (defined as “the strength of an individual´s belief that gender is a foundational force dictating a person´s characteristics”, p. 38) was associated with greater preference for a male primary breadwinner, as well as with work-family career tradeoffs that reinforce the gender wage gap. It has also been found that parents with stronger gender essentialist beliefs more strongly endorse gender prescriptions regarding appropriate jobs and activities for males and females, although this relation was not seen among their children [21].

Finally, GE beliefs have been found to both serve, and enhance, the system-justifying motive to believe that the social system, and thus the status quo, is just, fair, and good [22]. For example, both the threat of social change (in men) and activation of system-justification motives (in women and men) increase endorsement of essentialist accounts of gender differences [17, 23]. Conversely, Kray et al. [18] found that exposure to claims that gender roles are fixed, compared with presentation of information that they are changeable, increases gender system justification in men.

In short, existing research finds that facets of GE are associated with perceptions, behaviors, attitudes, and preferences that reinforce and justify the gender status quo. Here, using a broad new measure of GE and controlling for both political orientation and preference for social hierarchy (social dominance orientation), we expand on these findings. Scales used in previous research have often focused on specific components of GE, such as immutability [18, 20] and biological attributions [24], or specifically addressed parenting and parenthood rather than gender itself [19, 25]. Our new scale instead sought to make an additional contribution to the literature by incorporating the full breadth of this complex construct into a highly reliable measure of GE. Our scale is thus similar in intention to Rhodes & Gelman’s [26] 8-item scale and Park et al’s [27] 11-item scale, but somewhat longer (25 items), more comprehensive, and more psychometrically reliable. The earlier scales have demonstrated levels of reliability that are conventionally considered “acceptable”, with a mean α of .76 across the five studies in [27] and an α of .71 in [26], but a scale with higher reliability would be of value to some researchers.

We expected that people who regard men and women as distinctly, immutably, and naturally different (i.e., gender essentialists) would be less sympathetic to the erosion of gender role boundaries across both professional and personal domains, and in an organizational context would be more supportive of behaviors and attitudes that maintain gender inequalities. Thus, we examined relations between GE and discomfort with women and men taking on similar roles across five domains of adult life, support for discriminatory workplace practices, and the perception of contemporary workplaces as non-discriminatory. We hypothesized that GE would uniquely predict these dependent variables, independently of political orientation and preference for social hierarchy and inequality.

We also sought to investigate whether GE moderates an important form of organizational gender bias, the backlash effect. This effect, originally identified by Rudman [28], refers to negative characterization of individuals who violate gender norms, which may also result in negative consequences for the target such as economic or organizational penalties (e.g., [29]). It is thus a mechanism that contributes to the maintenance of gender roles. A primary focus of research has been backlash towards women who display stereotypically masculine traits such as agency or dominance (see [30, 31]). Research suggests that women who display such traits risk being perceived as being insufficiently communal (e.g., [32]). Furthermore, evidence suggests that this negativity stems at least in part from the violation of a gender *prescription*, rather than simply an expectation based on a descriptive norm. Thus, Okimoto and Brescoll [33] found that people were less supportive of a female politician who expressed power-seeking intentions, relative to male politicians and non-power-seeking females, due partly to feelings of moral outrage toward her.

Since belief in the ‘naturalness’ of gender stereotypical behavior is a component of GE thinking, there are reasons to hypothesize that those who hold GE beliefs will be more likely to express backlash. Griffiths [34], for example, has argued that essentialist thinking incorporates a normative element, whereby individuals who lack a supposedly essential trait–in this case, communality–are perceived to have deviated from their ‘intended’ developmental outcome. We therefore used Okimoto & Brescoll’s [33] experimental paradigm, superficially modified for our cultural and testing context, to test the hypothesis that those who hold GE beliefs will be more likely to express backlash, in the form of greater moral outrage and lower ratings of communality for power-seeking women in politics, as well as a disinclination to have power-seeking women as their own political candidates.

The study was conducted in two Western countries (Denmark and Australia), that both score relatively well on The Gender Inequality Index [35]. This captures three dimensions: reproductive health (measured by maternal mortality and adolescent birth rates), empowerment (measured by proportion of parliamentary seats held by women and proportion of adults with some secondary education), and economic status (measured by labor market participation). However, despite the relatively high gender equality ratings for both countries (which at time of testing was 5^th^ and 19^th^ for Denmark and Australia, respectively [36]) we nonetheless expected to observe a backlash effect, since this has been observed in other high-ranked countries (such as the United States, which currently ranks 43). Moreover, these dimensions are compatible with considerable sex-segregation of occupational roles, and prior research has found greater endorsement of gender stereotypes in developed countries scoring more favorably on such gender equality measures (e.g., [37, 38]).

## Materials and methods

The research was approved by the University of Melbourne Human Research Ethics committee (Ethics ID 1442956).

### Development of the Gender Essentialism Scale

Thirty-one candidate items for the Gender Essentialism Scale (GES) were written by the second and third authors based on previous conceptual analyses of essentialist thinking, and particularly those of Haslam et al. [2] and Rothbart and Taylor [3], which see it as understanding social categories as biologically grounded natural kinds. The authors decided that items should assess beliefs about the nature of the superordinate concept of gender rather than the nature of specific genders (i.e., women and men separately), the reference categories of some earlier measures (e.g., [2]). That decision was based on a desire for the GES to have broad applicability in the psychology of gender, the conviction that beliefs about the nature of gender are fundamentally relational or contrastive, and the judgment that it is more parsimonious to have a single gender essentialism scale than multiple scales assessing beliefs about different genders. By this reasoning, a scale assessing gender essentialism should therefore focus primarily on beliefs about and explanations for the nature of gender differences. Consequently, the large majority of items were framed comparatively, referring to women and men, girls and boys, or feminine and masculine. Some also contrasted mothers and fathers so as to address essentialist thinking in the context of particular gendered roles. Other items retained the focus on difference by referring to “each gender” or “sex differences.” Although items could have been written with an explicit reference to “gender” in the abstract, the authors believed that items framed in more concrete and contrastive ways would more directly capture people’s beliefs about gender difference.

Items were developed that aimed to tap several distinct components of essentialist thinking identified in previous work [2,3]. These includedthe belief that gender differences are discrete (e.g., “People tend to be either masculine or feminine; there’s not much middle ground”), biologically based and natural (e.g., “Differences between men and women are primarily determined by biology”), fixed or inalterable (e.g., “Differences between boys and girls are fixed at birth”), inherent (e.g., “Our gender is not just learned behavior: It is a deep part of who we are”), historically invariant (e.g., “In 100 years, society will think of the differences between women and men in much the same way as today”), and highly informative or “inductively potent” (e.g., “It is possible to know about many aspects of a person once you learn their gender”).

The initial set of 31 items, rated using a 5-point Likert scale ranging from 1 (strongly disagree) to 5 (strongly agree), were piloted with a sample of 154 Australian adults employed in the financial and legal service industries and the armed forces, who were participating in executive education programs. Analysis of item-total correlations, item variances, and verbal clarity led to the elimination of six items that failed to cohere, failed to discriminate among participants, or were confusing to participants. The final 25-item scale is presented in the supplementary information (S1 Appendix).

### Participants

The Danish survey firm YouGov, and its Australian partner, were contracted to sample participants from two different countries. A final total of 1814 survey panelists participated in the study, with 921 sampled from Australia (female = 451, male = 467, 3 did not indicate gender), and 893 sampled from Denmark (female = 444, male = 446, 3 did not indicate gender). A further 522 Australian and 281 Danish participants began the survey, but either chose not to continue after viewing the introductory page or were prevented from continuing due to failure to meet attentional checks (due to survey design, the breakdown of numbers falling into each category was not collected). Participants were recruited so that samples were nationally representative in gender, geography, and age, with the restriction that participants should be between the ages of 18–64. This recruiting strategy enables generalizability of findings across Western cultural boundaries and beyond predominantly young student samples. Participants also provided information about their personal income, although our samples were not nationally representative on this variable. Participants were paid in bonus points by the survey firms, which could then be cashed in for material items through the respective companies.

### Materials

Participants completed the 25-item GES, rated from 1 (strongly disagree) to 5 (strongly agree), and measures of sex-role egalitarianism, support for discriminatory practices, and perceived fairness of gender-based treatment. In addition, measures of political orientation and preference for social hierarchy were included to determine whether effects of the GES on the dependent variables were independent of these well-established predictors of endorsement of gender inequality.

To assess sex-role egalitarianism, participants completed the abbreviated Sex-Role Egalitarianism Scale (SRES), a 25-item questionnaire measured with a 5-point Likert scale, rated from 1 (strongly disagree) to 5 (strongly agree) [39]. The SRES assesses endorsement of sex equality of roles across five different domains of adult life: marital (e.g., “Cleaning up the dishes should be the shared responsibility of husbands and wives”), parental (e.g., “A husband should leave the care of young babies to his wife”, reversed), employment (e.g., “Women have as much ability as men to make major business decisions”), social-interpersonal-heterosexual (e.g., “A woman should be careful not to appear smarter than the man she is dating”, reversed), and educational (e.g., “Expensive job training should be given mostly to men”, reversed) (see also [40]).

Two short scales developed by Morton et al. [17], assessing support for discriminatory practices and perceived fairness of gender-based treatment, were also administered. The four-item Support for Discriminatory Practices scale (e.g., “If I were a manager in a company myself, I would believe that more often than not, hiring men is a better investment in the future of the company than hiring women”) and the 3-item Perceived Fairness of Gender-Based Treatment scale (e.g., “Discrimination against women in the labor force is no longer a problem in our country”), assess acceptance and denial, respectively, of continuing discrimination, each with a 7-point Likert scale rated from 1 (strongly disagree) to 7 (strongly agree).

Preference for social hierarchy was assessed with the Social Dominance Orientation Scale or SDO [41], a 16-item questionnaire with a 7-point Likert scale, rated from 1 (strongly disagree) to 7 (strongly agree). The SDO scale measures tolerance for hierarchical differences between social groups in general, soliciting agreement with statements such as “Some groups of people are simply inferior to other groups”. Political orientation was assessed by a single-item ranging from 1 (Very liberal) to 7 (Very conservative).

To measure the backlash effect, we utilized the paradigm reported in Okimoto and Brescoll [33]. The original vignettes were adapted for the Australian and Danish political contexts, but otherwise followed the original United States-based vignettes as closely as possible (material available from corresponding author on request). Participants were randomly assigned to read one of four vignettes, which provided a brief description and biography of a fictional political candidate. The backlash measure used a 2 (target gender) x 2 (target power-seeking behavior, present or absent) factorial design. Thus, two of the four vignettes described the political career of a male with aspirations in local politics, while two described the political career of a female with the same aspirations. Within both levels of the gender factor, one vignette described the target as high on power-seeking, while the other vignette did not include this characteristic.

After reading the vignette, participants were asked to rate the candidate, using 7-point Likert scales, on the dimensions ‘unsupportive-supportive’ and ‘uncaring-caring’. These were combined to create a measure of perceived communality. Participants also reported on a 7-point Likert scale the extent to which they felt contempt, anger, and disgust toward the candidate, these being combined to create a measure of moral outrage. Participants were also asked to report whether they would want the specific politician as their candidate on a rating scale from 1 (not at all) to 7 (very much), as a measure of candidate preference. These were the primary dependent variables for tesing the backlash effect and its moderation by GE.

In addition, participants rated the candidates using 7-point Likert scales on the dimensions ‘incompetent-competent’, ‘ineffective-effective’ and ‘unproductive-productive’ (combined to create a measure of competence), and ‘unassertive-assertive’, ‘weak-strong’, ‘not tough-tough’ (combined to create a measure of agency). The competence and agency measures were included for consistency with Okimoto and Brescoll (2010). Although they did not consider them as dimensions of backlash, they found an interaction between target gender and power-seeking for both, with power-seeking enhancing perceptions of only the male politician’s perceived competence and agency.

Thus we expected backlash to be evident on three measures–communality, moral outrage and candidate preference–demonstrated by statistical interactions between target gender and power-seeking behavior, such that female politicians would be penalized more for power-seeking behavior (seen as less communal, attracting more moral outrage, and less preferred as a candidate) relative to men. If GE plays a role in backlash then scores on the GES should moderate this interaction, so the backlash effect is stronger among more essentialist participants. As a secondary set of predictions, we also tested for an interaction between power-seeking and target gender for competence and agency, as well as moderation by GES.

### Procedure

The participants were invited to take part in a study of social attitudes and perceptions. All participants received the questionnaires and questions in the same order. These began with the backlash measure, for which there were two inclusion criteria: participants had to spend a minimum of 10 seconds reading the vignette, and correctly recall in which city the candidate lived. The survey automatically terminated for participants who spent fewer than 10 seconds reading the vignette. The backlash measures were followed by the political orientation item, the Gender Essentialism Scale, the Sex-Role Egalitarianism Scale, the Social Dominance Orientation Scale, then the Support for Discriminatory Practices and Perceived Fairness of Gender-based Treatment Scales. At the end of the questionnaire, Australian participants were asked to provide demographic information on gender (male, female, other–specify), age, region in the country, and personal income. For the Danish sample, YouGov already had this information for the participants, but the question of gender was asked in order to offer the option of “Other” for this part of the sample as well. Finally, all participants were offered the option to comment on the questionnaire to provide an opportunity for participants to express suspicion or other comments about the purpose of the study. This was the only question that could be left unanswered in the survey.

The Australian sample received all the questionnaires and the vignette in English, whereas all the stimulus materials were translated into Danish for the Danish participants, with all translations verified for meaning using back translation by a separate third party.

## Results

Scale means and reliabilities for the two samples are presented in Table 1. Most scales had very good to excellent internal consistency in both samples, with the partial exception of the Perceived Fairness of Gender-based Treatment scale, especially in the Australian sample. Notably the new GES had excellent reliability (α = 0.89 & 0.90) in both samples, substantially higher than the values obtained with shorter existing measures.

**Table 1:** Means, standard deviations, and scale reliabilities (Cronbach’s alpha) of the scales in the Australian and Danish samples. Note, SDP = Support for Discriminatory Practices; PFGT = Perceived Fairness of Gender-based Treatment; SDO = Social Dominance Orientation.

	Australia		Denmark	
	Mean (sd)	alpha	Mean (sd)	alpha
Gender Essentialism	3.08 (0.54)	.90	2.99 (0.49)	.89
Sex-role egalitarianism	3.93 (0.65)	.95	4.19 (0.58)	.95
SDP	2.37 (1.32)	.83	2.01 (1.18)	.84
PFGT	3.26 (1.23)	.60	3.84 (1.33)	.77
Political orientation	3.82 (1.33)	-	3.66 (1.21)	-
SDO	2.45 (1.15)	.94	2.79 (1.15)	.95
Communality	5.18 (1.30)	.90	4.62 (1.08)	.87
Moral outrage	2.25 (1.53)	.93	1.72 (1.14)	.96
Candidate preference	4.59 (1.38)	-	3.77 (1.41)	-
Agency	5.07 (1.22)	.88	4.46 (1.16)	.88
Competence	5.10 (1.33)	.95	4.53 (1.16)	.91

Men scored higher on the GES than women in Australia (*t*(916) = 4.02, *p* < .001) but there was no gender difference in Denmark (*t*(888) = 0.73, *p*>.05). In both samples, principal components analyses with Oblimin rotation and Kaiser normalization revealed two positively correlated components (for details see supplementary information, S1 and S2 Tables). In each case, a large majority of the 25 items (20 in Australia, 19 in Denmark) loaded substantially on the first component and the second component was dominated by the five reverse-scored items. These items constituted the entire second component in the Australian sample and three of them featured in the small second component in the Danish sample. Thus, aside from a weak methodological factor, the GES was substantively unidimensional in both samples, an interpretation that accords with the scale's high internal consistency and with the view that it indexes a coherent gender essentialism phenomenon. The internal consistency of the GES indicates that the scale captures one coherent phenomenon despite the items having somewhat diverse content (e.g., items tapping differences between women and men, and differences associated with particular gendered roles such as mothers and fathers).

As a prelude to a test of the GES’s capacity to predict the dependent variables (sex-role egalitarianism, support for discriminatory practices, and perceived fairness of gender-based treatment), independent of the control predictors (political orientation and social dominance orientation), the intercorrelations of these measures were examined. These correlations are presented in Table 2.

**Table 2:** Intercorrelations among gender essentialism and the criterion and control measures (Australian data above diagonal, Danish data below diagonal). Note, SDP = Support for Discriminatory Practices; PFGT = Perceived Fairness of Gender-based Treatment; SDO = Social Dominance Orientation.

	1	2	3	4	5	6
1. GE	-	-.50*	.40*	.35*	.28*	.40*
2. SRES	-.41*	-	-.74*	-.52*	-.28*	-.66*
3. SDP	.30*	-.64*	-	.54*	.23*	.66*
4. PFGT	.17*	-.23*	.27*	-	.17*	.48*
5. Political	.11*	-.08*	.09*	.01	-	.26*
6. SDO	.33*	-.49*	.50*	.34*	.12*	-

* *p* < .05

The table indicates that in both countries people scoring higher on GE tended to be less egalitarian regarding sex roles, more supportive of discriminatory treatment, more accepting of the fairness of gender-based treatment, somewhat more politically conservative, and more positively disposed toward social hierarchy. The relatively modest reliability of the PFGT, especially in the Australian sample, suggests that correlations between it and other scales should be interpreted with caution.

To determine whether GE made a unique predictive contribution to sex-role egalitarianism and the discrimination scales, after controlling for political orientation and preference for social hierarchy, a series of multiple regression analyses was performed. In each sample, each dependent variable scale was simultaneously regressed on GES, political orientation, and social dominance orientation. Table 3 presents the findings of these analyses. In each case, with the exception of perceived fairness of gender-based treatment in the Danish sample, the GES was independently associated with the dependent variables when political orientation and social dominance orientation were statistically controlled. Social dominance orientation had the strongest effects, consistent with its established status as a predictor of attitudes towards social inequality. The effects of political orientation were generally minimal and dwarfed by the effects of GE.

**Table 3:** Standardized beta weights from multiple regression analyses regressing sex-role egalitarianism (SRES), support for discriminatory practices (SDP), and perceived fairness of gender-based treatment (PFGT) on gender essentialism, social dominance orientation (SDO), and political orientation. Each column represents one analysis.

	Australia			Denmark		
Predictor	SRES	SDP	PFGT	SRES	SDP	PFGT
GE	-.27**	.16**	.18**	-.28**	.15**	.06
Political orientation	-.07**	.04	.01	.00	.02	-.04
SDO	-.53**	.58**	.41**	-40**	.45**	.33**

** *p* < .01

* *p* < .05

### Backlash effect

Backlash effects were examined through a series of linear models, with target (politician) gender (male or female), power-seeking behavior (present or absent), and GES scores as predictors. Four-way analyses that included participant gender as a factor were also conducted, but as this factor yielded no significant main or interaction effects for any backlash measure in either sample, the simpler three-way analyses are reported instead. Separate analyses were conducted for the three backlash-relevant measures: perceived communality of the politician, moral outrage at the politician, and support for the politician as the participant’s candidate. Backlash would be demonstrated by target Gender × Power-seeking interactions in the expected direction, and moderation of backlash by GE would be demonstrated if these two-way interactions were accompanied by significant three-way target Gender × Power-seeking × GE interactions.

Significant backlash effects were found in the Australian sample for moral outrage and in the Danish sample for moral outrage and candidate preference (see Tables 4 and 5). In addition to the predicted backlash effect, the Australian moral outrage analysis also found significantly greater outrage towards male politicians, towards low power-seeking politicians, and among participants higher in gender essentialism, as well as significant two-way interactions between essentialism and both target gender and power-seeking (i.e., less outrage towards men and power-seeking political candidates among essentialists). The Danish moral outrage analysis also showed greater outrage towards low power-seeking politicians, among gender essentialists, and a significant interaction between these two factors (i.e., less outrage among essentialists towards low power-seekers). Finally, in addition to the predicted backlash effect, the Danish candidate preference analysis showed greater preference for candidates who were power-seeking and among gender non-essentialists, and significant interactions between essentialism and both target gender and power-seeking (i.e., essentialists showed greater preference than non-essentialists for candidates who were male and power-seeking).

**Table 4:** Summary of linear models for the backlash and related measures in the Australian sample.

	Communality	Moral outrage	Candidate preference	Agency	Competence
Intercept	4.46***	-1.11	3.88***	3.59***	3.80***
Gender	0.46	2.15*	1.01	1.39*	0.88
Power seeking	-0.58	3.09***	-0.54	0.15	-0.09
GE	0.21	1.02***	0.19	0.40*	0.39*
Gender x Power	0.96	4.13***	1.19	0.40	0.57
Gender x GE	-0.11	-0.64*	-0.29	-0.39	-0.24
Power x GE	0.21	-0.93***	0.20	0.06	0.05
Gender x Power x GE	-0.34	1.27***	-0.35	-0.14	-0.20

*** *p* < .001

** *p* < .01

* *p* < .05

**Table 5:** Summary of linear models for the backlash and related measures in the Danish sample.

	Communality	Moral outrage	Candidate preference	Agency	Competence
Intercept	5.36***	0.29	5.41***	4.50***	5.51***
Gender	-0.69	0.91	-1.33	-0.10	-1.14
Power-seeking	-0.97	1.60*	-2.37**	0.23	-1.19
GE	-0.25	0.48**	-0.60**	-0.12	-0.42**
Gender x Power	1.35	-1.86*	2.77*	1.08	2.31
Gender x GE	0.27	-0.38	0.52*	0.03	0.44*
Power x GE	0.30	-0.48*	0.86**	0.12	0.55*
Gender x Power x GE	-0.47	0.67*	-1.00*	-0.33	-0.82**

*** *p* < .001

** *p* < .01

* *p* < .05

**Fig 1:**
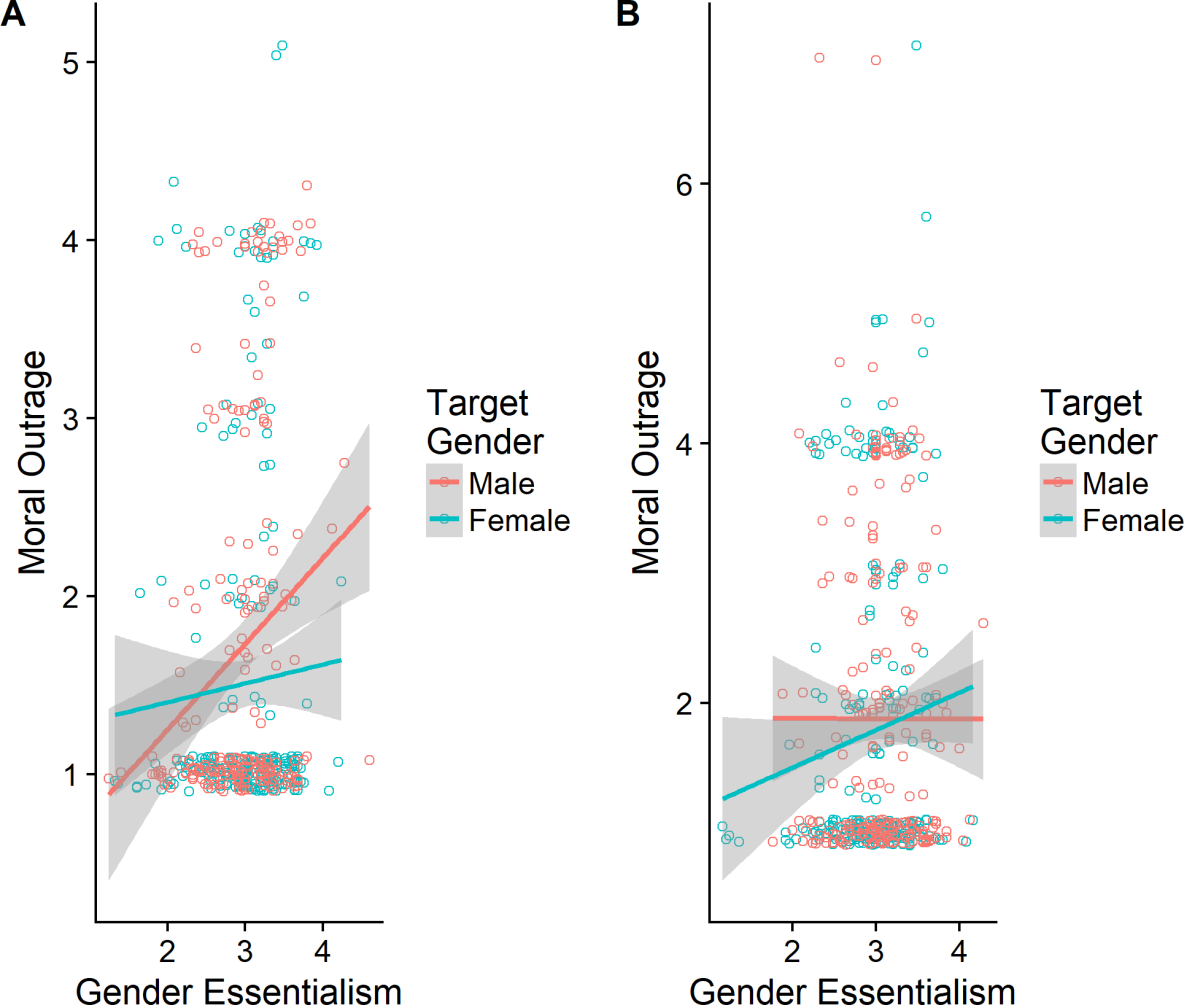
The effects of gender essentialism and target gender on moral outrage ratings. The figure represents the data points with .1 jitter added to avoid overplotting, the fitted linear trend for each condition, and 95% confidence intervals.

**Fig 2:**
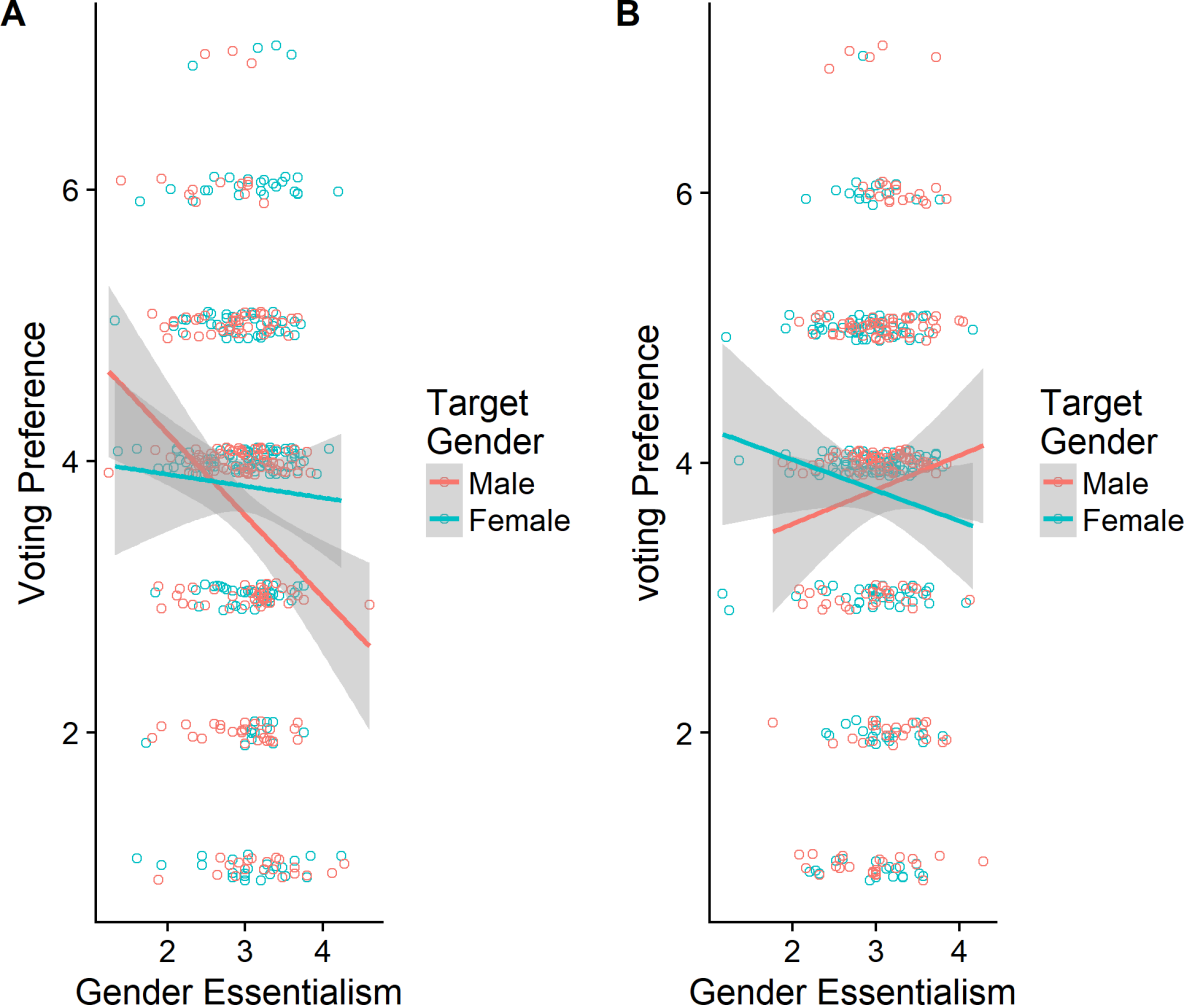
The effects of gender essentialism and target gender on voting preferences. The figure represents the data points with .1 jitter added to avoid overplotting, the fitted linear trend for each condition, and 95% confidence intervals.

Each of the three backlash effects was significantly moderated by GE. (All of these moderation effects remained in re-analyses where GE was mean-centered for each participant based on the candidate type they rated. See supplementary information, S3 and S4 Tables, for these analyses.) For illustration, we show the moderation effects for moral outrage (Fig 1) and candidate preference (Fig 2), which present the low and high power-seeking conditions in panels A and B, respectively. Post hoc linear model analyses restricted to participants in each condition showed significant target Gender × GE interactions in both low and high power-seeking conditions (in different directions) in the Australian moral outrage analysis: low power-seeking, *β* = -0.64, *t =* -2.43, *p* = .016; high power-seeking, β = 0.63, *t =* 2.54, *p* = .012.

As Fig 1 shows, in the high power-seeking condition, higher GE is associated with greater moral outrage toward female but not male candidates, whereas the opposite pattern occurs in the low-power-seeking condition. By implication, gender essentialists are inclined to object to power-seeking women and non-power-seeking men. This finding is novel as it points to an unexpected backlash effect against the non-power-seeking men in addition to the expected backlash against power-seeking women. Fig 2 presents the trends for candidate preferences in the Danish sample. In this case, gender essentialists prefer the male candidate to the female candidate when they are power-seeking, but the female candidate to the male candidate when they are not. Post hoc analyses indicated that the corresponding target Gender × GE interactions were marginal in the high power-seeking condition, β = -0.48, *t =* -1.67, *p* = .095; but significant in the low power-seeking condition, β = 0.52, *t =* 1.98, *p* = .048. Again, the overall pattern indicates a backlash reaction against the female candidate when she is presented as seeking power, especially for participants high in gender essentialism.

Although not considered backlash effects, we also tested for two- and three-way interactions for competence and agency. We found no such interactions in the Australian sample, nor for agency in the Danish sample (see Tables 4 and 5). However, significant two- and three-way interactions for competence were identified for Danish participants. Linear models showed significant effects of high vs low power-seeking both for the male candidate (β = .44, *t =* 4.12, *p <* .05) and the female candidate (β = .33, *t =* 3.15, *p <* .05), although inspection of means suggested this effect was larger for the male politician (Mean [high power] = 4.72, SD = 1.29; vs Mean [low power] = 4.27, SD = .96;) than the female (Mean [high power] = 4.75, SD = 1.05; vs Mean [low power] = 4.41, SD = 1.24). Inspection of trends showed that gender essentialists perceive the male candidate to be more competent when he was power seeking, but less competent when he was not, once again indicating a backlash against the non-normative male candidate.

It is possible that any associations between gender essentialism and other variables in the backlash analysis might partially reflect priming, given that participants were exposed to different political candidates (either a non-power seeking male, a power-seeking male, a non-power-seeking female, or a power-seeking female) prior to completing the GES. If this were the case, we would expect participants who were exposed to stereotypical candidates (the power-seeking male and the non-power-seeking female) to score higher on gender essentialism. However, a 2 (non-power-seeking vs. power-seeking) x 2 (male vs. female) between-subjects ANOVA on the Australian sample´s GE scores revealed no main or interaction effects (all *p*> 0.05), indicating that no priming occurred. The same analysis on the Danish sample´s GE scores revealed no main effects (both *p*> 0.0.5) but a significant two-way interaction F(1,889) = 6.10, p = 0.014. However, this relatively weak effect, where participants exposed to stereotypic candidates scored slightly higher on the GES than those exposed to non-stereotypic candidates (3.03 vs. 2.95), could not have accounted for GE moderating the backlash effects, as these interactions involve not the mean level of GE for different targets but GE’s association with other variables (e.g., outrage) for specific targets, as illustrated in Figs 1 and 2.

## Discussion

Gender essentialists regard gender differences as natural, fixed, deep-seated, discrete, informative, and fundamental, beliefs that often rest on a biogenetic understanding of the sources of gender difference. Popular discourse often presents such ‘essential’ differences as an argument for greater progress toward gender equality, the rationale being that distinctive and complementary masculine and feminine skills and perspectives are both required in critical decision-making processes. The current study, however, adds to existing evidence that GE thinking is associated with support for the gender status quo.

Our new scale, examined for the first time here using two large and diverse samples from Denmark and Australia, provides the most comprehensive measure of the complex construct of gender essentialism to date. It was very highly reliable in both Australia and Denmark, achieving levels of internal consistency (0.89–0.90) that substantially exceed those of earlier scales (0.71–0.76), probably in part because it is a longer scale, and is therefore likely to improve predictive validity in research studies. The factor-analytic evidence and the high internal consistency indicate that GES items assess a coherent set of beliefs about gender.

As expected, and providing a conceptual replication and expansion of previous findings [18], higher scores on the GES were associated with less egalitarian views of sex roles covering five important domains of adult life: marital; parental; educational; employment; and social-interpersonal-heterosexual. Crucially, the association between the GES and these dependent variables were independent of the effects of political orientation or a generalized preference for social inequality. In the only comparable prior work, using a scale assessing belief in the fixedness of gender roles rather than gender essentialism more broadly, Kray and colleagues [18] showed that their measure had predictive validity over and above political orientation, but not social dominance orientation, which commonly has greater predictive potency. Moreover, our study expanded existing evidence of an association with support for traditional sex roles (the Traditional Egalitarian Sex Roles scale [42]) to demonstrate such an association in a measure that explicitly provides a comprehensive representation of important adult roles across five different domains.

We also found that GE, as measured by our scale, predicted greater support for gender discriminatory practices in both countries, and perceived fairness of the differential treatment of women and men in Australia. Our findings therefore indicate that GE, as measured by the GES, makes a unique predictive contribution to two forms of gender bias. These findings empirically expand the known correlates of GE in a novel but expected direction. Prior arguments that essentialist thinking is associated with endorsement of inequalities between groups are supported by our findings that gender essentialists are more accepting of workplace gender discrimination, while simultaneously perceiving that such discrimination is not prevalent, and reporting greater discomfort with the erosion of the boundaries of traditional gender roles.

Our findings only partially replicated previous work on backlash, demonstrating significant backlash on one of three measures in the Australian sample and two of three in the Danish sample. However, whenever backlash effects emerged they were moderated in the expected way by GE. In the Australian sample, GES scores predicted greater differential moral outrage against power-seeking female relative to male political candidates. This finding was replicated in the Danish sample, where GES scores were also associated with greater backlash on the dimension of lower preference for power-seeking female candidates. GES scores also moderated the differential effect of power-seeking on perceived competence in the Danish sample. Why there should be more evidence of backlash effects in Denmark than in Australia, and more evidence that individual differences in GE are implicated in judgments of political candidates, is uncertain. Denmark ranks as more equal on the Gender Inequality Index and the Danish sample tended to be somewhat more egalitarian and less supportive of gender discrimination than the Australian sample. On the other hand, better scores on gender equality indices that tap factors such as health, educational access and economic and political empowerment do not necessarily correspond to lower endorsement of gender stereotypes or less occupational sex-segregation[43, 44]. It is sobering that backlash is still evident in a country considered to be one of the world’s most gender equal, and that GE is implicated in inegalitarian judgments there.

Contrary to previous research, no backlash effect was obtained for perceived communality of the candidates, ruling out the possibility of finding a role of GE in such an effect. This may have been due to our choice of backlash testing context. Meta-analytic reviews find that the backlash effect is small [45], and depends on expressions of dominance being explicit rather than implicit [31]. Another meta-analytic review suggests that backlash may depend on specific aspects of the task and the sample. Thus, Koch, D’Mello and Sackett [46] recently found no evidence of backlash against competent women, and showed that students and working adults were more susceptible to gender role incongruity than experienced professionals. Potentially, a local politician in small population countries like Australia and Denmark may not be considered as powerful as a state senator in the United States (the context used by [33]). It is possible this may also explain why we largely did not replicate their findings of an interaction between power-seeking and politician gender for perceived competence and agency in our samples (the exception being competence in the Danish sample, which was moderated by GE). A goal for future research would therefore be to look at relations between GE and backlash involving responses to more explicit or salient portrayals of dominance.

An interesting and unexpected finding from the backlash analyses was that gender essentialists not only evaluated power-seeking women more negatively than men, but also tended to respond more negatively to non-power-seeking men. This finding suggests that gender essentialism underpins or magnifies responses to violations of gender norms in general, whether these are displayed by women or men, and by commission of proscribed behaviors or by omission of prescribed behaviors. Although our findings show that gender essentialism is associated with acceptance of social arrangements that disadvantage and harm women, such as support for discriminatory hiring practices and unequal gender roles, it may also be associated with negative assessments of men who fail to abide by masculine norms. If gender essentialism supports an unequal gender status quo for those who adhere to gender norms and amplifies backlash against women and men who do not adhere to them, then its only real beneficiaries are norm-adhering men.

We had not hypothesized that there would be any significant cultural differences between Australia and Denmark on our measures or findings. However, one unexpected finding of difference (additional to those reported above) was that Australian men were more politically conservative and scored higher on the GES than Australian women, whereas there were no significant differences between the genders in the Danish sample. This difference points to non-trivial cultural differences between even these two relatively gender-equal countries, as well as scope for patterns to vary across time and place.

The finding that GE thinking is associated with support for discrimination and inequality has important implications for gender equality advocates and practitioners. Many academics have drawn attention to the essentialist implications of ‘business case’ arguments for greater senior female representation (e.g., [11, 47]). Arguments for political quota systems may also encourage essentialist thinking, “by suggesting that for essentialist reasons, only women can represent women (and therefore that women cannot represent men).” ([48], p.631). Our findings provide evidence for potential adverse repercussions of such implications, and thus the critical need for care and accuracy in how such arguments are made.

Our findings also have implications for organizations seeking to increase gender equality. Currently, many organizations are making strenuous efforts to reduce gender bias through programs on unconscious bias [49], despite apparently limited effectiveness [50]. Moreover, such training focuses largely on the biasing effects of *descriptive* aspects of gender stereotypes on evaluations, and thus will largely fail to address the negatively charged moral emotions evoked by agentic women (see [51]). Our findings provide preliminary evidence that a focus on challenging inaccurate GE beliefs, which may contribute to gender prescriptions, could potentially be a valuable alternative to unconscious bias training programs (see also [21]).

Psychological research is increasingly revealing the role of gender essentialism in promoting and enabling gender inequality. The present study strengthens this case further, demonstrating that this coherent and rarely challenged set of beliefs is a basis for tolerance of inequality and discrimination. Gender essentialism is a potentially critical target for research and intervention, and the GES is a promising instrument for capturing it.

## Supporting information

S1 Appendix The gender essentialism scale. (DOCX)

S1 Table Principal components analysis for the Australian sample. (DOCX)

S2 Table Principal components analysis for the Danish sample. (DOCX)

S3 Table Summary of linear models for backlash and related measures in the Australian sample, with GE mean centred within conditions. (DOCX)

S4 Table Summary of linear models for backlash and related measures in the Danish sample, with GE mean centred within conditions. (DOCX)
