# Supplementary material for: Beyond Mars and Venus: The role of gender essentialism in support for gender inequality and backlash
Source: PLoS One. 2018 Jul 24;13(7):e0200921. doi: 10.1371/journal.pone.0200921 (PMC6057632; doi:10.1371/journal.pone.0200921)
Supplement: S1 Appendix — (DOCX) [file pone.0200921.s001.docx]

**S1 Appendix. The Gender Essentialism Scale**

1 Differences between women and men’s personalities are in their DNA.

2 Men and women have different abilities

3 Genes are at the root of differences between the sexes

4 People generally over-estimate how much sex differences in behaviour are biologically based (R)

5 Differences between men and women in behaviour and personality are largely determined by genetic predisposition.

6 Fathers have to learn what mothers are able to do naturally

7 People tend to be either masculine or feminine: there’s not much middle ground

8 Wherever you go in the world, men and women differ from one another in the same kinds of ways.

9 Members of each gender have many things in common (R)

10 It is possible to know about many aspects of a person once you learn their gender.

11 Trying to make boys and girls have similar likes and dislikes is pointless.

12 In 100 years, society will think of the differences between women and men in much the same way as today.

13 Women and men are fundamentally different.

14 Women are innately more nurturing than men

15 Knowing that someone is a man tells you very little about what the person is like (R)

16 Men and women’s personalities are more or less the same (R)

17 Men and women differ in numerous ways.

18 Their underlying nature makes it difficult for men to learn to behave more like women.

19 Differences between boys and girls are fixed at birth

20 Mothers are naturally more sensitive to a baby’s feelings than fathers are.

21 Men and women have different personality types

22 Male and female brains probably work in very different ways

23 Differences between men and women are primarily determined by biology

24 Women are naturally less aggressive than men.

25 Upbringing by parents and the social environment have far greater significance for the development of sex differences than inborn differences in female and male brains (R)
