# Supplementary material for: Beyond Mars and Venus: The role of gender essentialism in support for gender inequality and backlash
Source: PLoS One. 2018 Jul 24;13(7):e0200921. doi: 10.1371/journal.pone.0200921 (PMC6057632; doi:10.1371/journal.pone.0200921)
Supplement: S1 Table — (DOCX) [file pone.0200921.s002.docx]

**S1 Table. Principal Components Analysis for the Australian sample**

| GE Questions | Component 1 | Component 2 |
| --- | --- | --- |
| Women are naturally less aggressive than men | 0.57 |  |
| Women are innately more nurturing than men | 0.69 |  |
| Women and men are fundamentally different | 0.72 |  |
| Wherever you go in the world, men and women differ from one another in the same kinds of ways | 0.62 |  |
| Trying to make boys and girls have similar likes and dislikes is pointless | 0.51 |  |
| Their underlying nature makes it difficult for men to learn to behave more like women | 0.75 |  |
| People tend to be either masculine or feminine: there’s not much middle ground | 0.64 |  |
| Mothers are naturally more sensitive to a baby’s feelings than fathers are | 0.65 |  |
| Men and women have different personality types | 0.71 |  |
| Men and women have different abilities | 0.68 |  |
| Men and women differ in numerous ways | 0.56 |  |
| Male and female brains probably work in very different ways | 0.64 |  |
| It is possible to know about many aspects of a person once you learn their gender | 0.64 |  |
| In 100 years, society will think of the differences between women and men in much the same way as today | 0.56 |  |
| Genes are at the root of differences between the sexes | 0.65 |  |
| Fathers have to learn what mothers are able to do naturally | 0.61 |  |
| Differences between women and men’s personalities are in their DNA | 0.70 |  |
| Differences between men and women in behaviour and personality are largely determined by genetic predisposition | 0.69 |  |
| Differences between men and women are primarily determined by biology | 0.69 |  |
| Differences between boys and girls are fixed at birth | 0.71 |  |
| GE Upbringing by parents and the social environment have far greater significance for the development of sex differences than inborn differences in female and male brains (R) |  | 0.68 |
| People generally over-estimate how much sex differences in behaviour are biologically based (R) |  | 0.45 |
| Men and women’s personalities are more or less the same (R) |  | 0.58 |
| Members of each gender have many things in common (R) |  | 0.54 |
| Knowing that someone is a man tells you very little about what the person is like (R) |  | 0.56 |
