# Supplementary material for: Beyond Mars and Venus: The role of gender essentialism in support for gender inequality and backlash
Source: PLoS One. 2018 Jul 24;13(7):e0200921. doi: 10.1371/journal.pone.0200921 (PMC6057632; doi:10.1371/journal.pone.0200921)
Supplement: S3 Table — (DOCX) [file pone.0200921.s004.docx]

**S3 Table. Summary of linear models for backlash and related measures in the Australian sample, with GE mean centred within conditions**

|  | Communality | Moral outrage | Candidate preference | Agency | Competence |
| --- | --- | --- | --- | --- | --- |
| Intercept | 5.11*** | 2.03*** | 4.46*** | 4.81*** | 5.01*** |
| Gender | 0.13 | 0.17 | 0.12 | 0.22 | 0.12 |
| Power-seeking | 0.09 | 0.26 | 0.10 | 0.36** | 0.08 |
| GE | 0.21 | 1.02*** | 0.19 | 0.40* | 0.40* |
| Gender x Power | 0.21 | -0.20 | 0.11 | -0.04 | -0.06 |
| Gender x GE | -0.11 | -0.64* | -0.29 | -0.39 | -0.24 |
| Power x GE | 0.21 | -0.93*** | 0.20 | 0.06 | 0.05 |
| Gender x Power X GE | -0.33 | 1.27*** | -0.35 | -0.14 | -0.19 |

*** *p* < .001

** *p* < .01

* *p* < .05
