# Supplementary material for: Beyond Mars and Venus: The role of gender essentialism in support for gender inequality and backlash
Source: PLoS One. 2018 Jul 24;13(7):e0200921. doi: 10.1371/journal.pone.0200921 (PMC6057632; doi:10.1371/journal.pone.0200921)
Supplement: S4 Table — (DOCX) [file pone.0200921.s005.docx]

**S4 Table. Summary of linear models for backlash and related measures in the Danish sample, with GE mean centred within conditions**

|  | Communality | Moral outrage | Candidate preference | Agency | Competence |
| --- | --- | --- | --- | --- | --- |
| Intercept | 4.61*** | 1.72*** | 3.61*** | 4.14*** | 4.25*** |
| Gender | 0.11 | -0.21* | 0.20 | 0.00 | 0.16 |
| Power-seeking | -0.08 | 0.16 | 0.19 | 0.60*** | 0.46*** |
| GE | -0.25 | 0.48** | -0.60** | -0.11 | -0.42** |
| Gender x Power | -0.05 | 0.13 | -0.21 | 0.09 | -0.13 |
| Gender x GE | 0.27 | -0.38 | 0.52* | 0.03 | 0.44* |
| Power x GE | 0.30 | -0.48* | 0.86** | 0.12 | 0.55* |
| Gender x Power X GE | -0.47 | 0.67* | -0.99* | -0.33 | -0.82** |

*** *p* < .001

** *p* < .01

* *p* < .05
